# Efficient Removal of Cationic Dye by Biomimetic Amorphous Calcium Carbonate: Behavior and Mechanisms

**DOI:** 10.3390/molecules29225426

**Published:** 2024-11-18

**Authors:** Renlu Liu, Weizhen Ji, Jie Min, Pengjun Wen, Yan Li, Jialu Hu, Li Yin, Genhe He

**Affiliations:** Key Laboratory of Jiangxi Province for Functional Biology and Pollution Control in Red Soil Regions, School of Life Sciences, Jinggangshan University, Ji’an 343009, China; liurenlu89@163.com (R.L.); jiweizhen0224@163.com (W.J.); 19179682360@163.com (J.M.); wpj01172005@163.com (P.W.); ly_xmx5077@163.com (Y.L.); huluoluoluo@163.com (J.H.); yinli_voyage@126.com (L.Y.)

**Keywords:** biomimetic materials, amorphous calcium carbonate, cationic dye, adsorption, mechanisms

## Abstract

The search for efficient, environmentally friendly adsorbents is critical for purifying dye wastewater. In this study, we produced a first-of-its-kind effective biomimetic amorphous calcium carbonate (BACC) using bacterial processes and evaluated its capacity to adsorb a hazardous organic cationic dye—methylene blue (MB). BACC can adsorb a maximum of 494.86 mg/g of MB, and this excellent adsorption performance was maintained during different solution temperature (10–55 °C) and broad pH (3–12) conditions. The favorable adsorption characteristics of BACC can be attributable to its hydrophobic property, porosity, electronegativity, and perfect dispersity in aqueous solution. During adsorption, MB can form Cl-Ca, S-O, N-Ca, and H-bonds on the surface of BACC. Since BACC has excellent resistance to adsorption interference in different water bodies and in real dye wastewater, and can also be effectively recycled six times, our study is an important step forward in dye wastewater treatment applications.

## 1. Introduction

Dyes are organic chemicals characterized by their unique conjugated structures, which have been extensively used for coloring textile fibers, leather, and paper [1,2]. As a result, dyes are the most common organic pollutant in industrial effluents across various sectors, including textiles, food processing, rubber, cosmetics, and pharmaceuticals [3]. One cationic dye, methylene blue (MB), is a commonly employed organic cationic dye that readily dissolves in water and possesses inherent toxicity, leading to negative impacts on ecosystems and potential adverse health effects in humans such as nausea, vomiting, or respiratory distress [4,5,6]. Thus, the pre-treatment of methylene blue in water is essential for protecting human and environmental health. Various technologies can be used to treat organic dye effluents, including physical methods such as ion exchange, adsorption, and membrane filtration, chemical methods such as photo-Fenton and coagulation–flocculation, and biological methods such as bacteria degradation, algae degradation, and enzyme treatments [7,8]. However, these methods are frequently limited by various drawbacks, including high costs, poor reusability, low efficacy, and excessive sludge production. Adsorption is widely recognized as the most effective method for removing dye contaminants from water bodies due to its technical simplicity and ease of operation [9,10]. Currently, biochar, activated carbon, hydrogels, zeolites, and alumina are commonly used to eliminate organic dye molecules from effluent [4,8,11]. However, existing adsorbent materials have several defects such as low adsorption capacity, poor dispersion, high preparation cost, and unstable application effects due to environmental factors, which make it difficult to popularize their application on a large scale. Therefore, there is an urgent requirement to develop cost-effective, eco-friendly, and efficient adsorbent materials.

Biomineralization synthesis emerges as a promising alternative, offering a green, energy-efficient strategy by using mild conditions such as ambient temperature and pressure, as well as near-neutral solution environments [12,13]. Recently, biomimetic materials have been recognized as effective materials for environmental remediation due to their unique organic–inorganic composite structural characteristics, and living organisms are often capable of forming minerals with exquisite morphologies and uniquely assembled superstructures, which have more exposed adsorption sites and thereby enhance pollution removal and purification. Calcium carbonate is a typical mineral widely found in nature and biological systems, with a complex structure and unique functions, and is also used as one of the ideal models in biomimetic materials research [12,14]. The main known forms of calcium carbonate are calcite, calcite monohydrate, calcite hexahydrate, aragonite, vaterite, calcium carbonate hemihydrate, and amorphous calcium carbonate (ACC), of which calcite is the most thermodynamically stable form [15,16]. Biomimetic amorphous calcium carbonate (BACC) is thermodynamically unstable and is often the product of an intermediate state of the reaction, easily transforming into other stable phases. In chemical synthesis, organic or inorganic additives are often added to stabilize ACC. However, the preparation process is marred by inefficiencies, difficulties in achieving pure ACC, and environmental sensitivities [17]. Therefore, preparing stabilized ACC using biological methods is of great value to its further application and for deepening understanding of its environmental benefits.

Most microbes in the environment are capable of inducing the synthesis of calcium carbonate minerals and modulating the conformation of calcium carbonate minerals to synthesize stable BACC [12,18]. Compared with the inorganic chemical mineralization process, microbial-mediated synthesis of calcium carbonate typically results in nanoscale structures with porous surfaces and a well-developed mesoporous configuration which exhibits excellent purification capabilities for inorganic pollutants (heavy metals) [12,19,20]. BACC, a very important calcium carbonate mineral of biogenic origin, is often overlooked in its effect on the transport and transformation of pollutants due to its amorphous nature in the environment. Given that no environmental significance study has been conducted to date, it is essential to know the ecological effects, mechanism of action, and adsorption properties of BACC on cationic organic dye. *Bacillus licheniformis* (*B. licheniformis*) is prevalent in the environment, with exceptional adaptability [21,22]. It can induce substantial BACC synthesis in liquid culture. MB was selected as a dye due to its stability in common reduction and oxidation processes and the difficulty in removing it from industrial wastewater.

We, for the first time, explored the effects of microbially induced synthesized BACC on the purification of MB in wastewater and its mechanism of action, to further understand the environmental significance of BACC, which not only helps elevate the technology level of dye wastewater treatment, but also broadens the horizons for research on the environmental and biological self-purification theoretical system.

## 2. Results and Discussion

### 2.1. Characterization of the BACC

**Figure 1:**
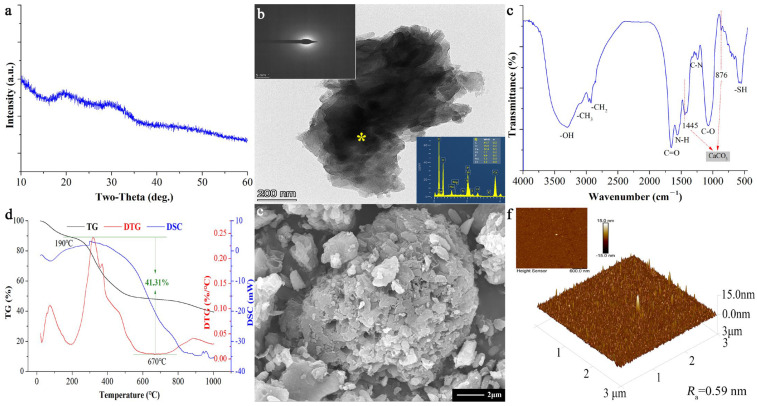
Structural and morphological characteristics of tested BACC. XRD pattern (**a**), TEM-SAED-EDS (**b**), FTIR spectra (**c**), TG-DTG/DSC results (**d**), SEM image (**e**), and 3D-AFM image of BACC (**f**).

The XRD pattern (Figure 1a) of biominerals shows no obvious diffraction peaks, which is consistent with the absence of diffraction rings or spots observed in the TEM-SAED analysis (Figure 1b). Elemental analysis through energy-dispersive X-ray spectroscopy (EDS) reveals that the main elements are C, O, and Ca, suggesting that these biominerals are primarily ACC [12,23,24,25]. Organic functional groups such as -OH, -CH_3_, -CH_2_, C=O, N-H, C-N, C-O, and -SH are detected at 3383, 2964, 2927, 1658, 1516, 1240, 1076, and 579 cm^−1^ in the FTIR spectrum (Figure 1c) of BACC alongside ACC [26,27]. Thermogravimetric analysis (TGA) coupled with derivative thermogravimetry (DTG) and differential scanning calorimetry (DSC) indicates that the mass loss of BACC occurs in three stages (Figure 1d). The first stage showed a 10.78 wt% loss from 25 °C to 190 °C attributed to water evaporation [26,28]. In agreement with the findings of the FTIR investigation, the mass loss in the second stage (41.31 wt% loss) occurred from 190 °C to 670 °C, which is mainly caused by the decomposition and combustion of organic matter in BACC [12]. The final stage involved an 8.63 wt% loss from 670 °C to 1000 °C due to the decomposition of CaCO_3_ into CaO and CO_2_ [26,28]. Collectively, these analyses support the characterization of BACC as an organic–inorganic mineral composite material, with an organic matter content of about 41.31 wt%. These organic matters are essential for maintaining the stability of the BACC structure [12,29].

The morphology of BACC was investigated by SEM. The biominerals are composed of aggregates of irregularly shaped, porous particles (Figure 1e). Additionally, the 3D-AFM showed a tightly aggregating micro- and nano-porous structure, with a surface roughness quantified by the arithmetic average roughness value (*R*_a_) of 0.59 nm (Figure 1f). The AFM results are consistent with the SEM images (Figure 1e).

### 2.2. Adsorbent Dosage Effect

**Figure 2:**
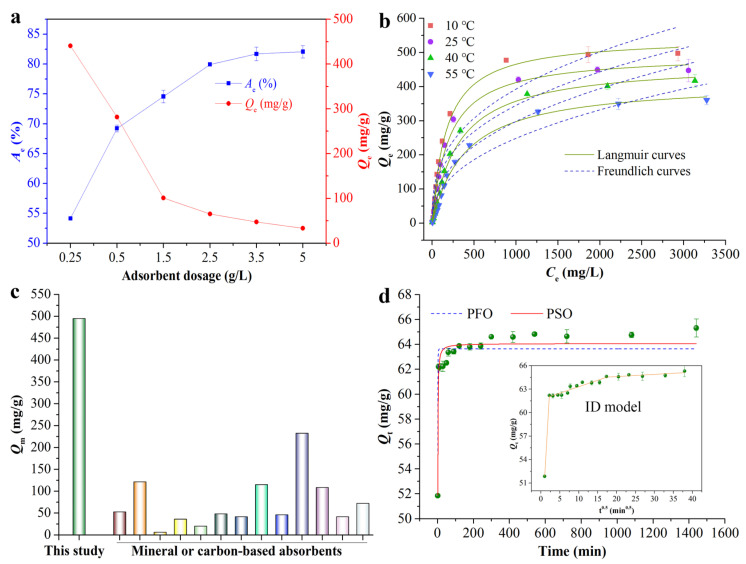
Effect of adsorbent dose (**a**) and adsorption isotherm at different temperatures on MB adsorption by BACC fitted to Langmuir and Freundlich models (**b**), comparison of Qm between BACC and reported mineral or carbon-based adsorbents from Table S1 (**c**), adsorption as a function of contact time fitted to PFO, PSO (**d**), and ID models (**d**), (insert). Data represent the mean ± standard deviation (s.d.) from three independent experiments.

The MB adsorption efficacy of BACC is influenced by the adsorbent dosage, as illustrated in Figure 2a. With an increase in the quantity of BACC (<2.5 g/L), the adsorption process’s efficiency was enhanced. This may result from an increase in the adsorbent’s specific surface area. The MB adsorption efficiency of BACC peaked at a dosage of 2.5 g/L. Adsorption efficiency remained mostly unchanged when additional BACC was added above this concentration. Figure 2a shows that as the amount of BACC increased, the *Q_e_* decreased due to the adsorption reaction involving more unsaturated adsorption sites [30]. Another reason could be that high adsorbent concentrations tend to aggregate, which reduces the total surface area of BACC and extends the diffusion path [4,31]. Other adsorbents have also exhibited similar features of MB adsorption [27,32].

### 2.3. Adsorption Isotherm and Thermodynamic Analysis

The analysis of adsorption isotherms for BACC and ACC offers insights into adsorption mechanisms. With an increase in MB concentration in the equilibrium solution (*C_e_*), the *Q_e_* also increased (Figure 2b). The color of BACC turned navy blue after adsorption (Figure S2), indicating strong MB adsorption stability. The empirical data were analyzed using the Freundlich and Langmuir adsorption models to understand the adsorption dynamics. The Langmuir model (R^2^~0.99, Adjusted R-squared (ARE)~0.99) better described the MB adsorption by BACC at 10, 25, 40, and 55 °C than the Freundlich model (R^2^~0.90, ARE~0.88) (Figure 2b and Table 1), suggesting a constant adsorption site energy and a maximum coverage of the monolayer surface [19,33]. Therefore, the capacity for MB adsorption is heavily influenced by the surface microstructure of BACC. According to Table 1, the *Q_m_* of BACC for MB was 544.87, 494.86, 470.86, and 414.55 mg/g at 10, 25, 40, and 55 °C, respectively. The *Q_m_* for MB increased as the temperature decreased, suggesting that BACC will be more effective in treating MB wastewater in cold climate regions, such as northern China or southern China in the winter. Compared to other widely used mineral or carbon-based adsorbents, the *Q_m_* of BACC (with a simple preparation method) for MB was significantly greater (Figure 2c and Table S1). Although the *Q_m_* of some adsorption materials (such as KOH-activated carbon [34], graphene oxide/magnesium oxide nanocomposites [35], etc.) for MB is greater than that of BACC, the preparation process of these materials is too complex, environmentally unfriendly, and costly, making them unsuitable for large-scale promotion and application. In addition, BACC has a better adsorption capacity than APCC (chemically synthesized calcium carbonate) and widely available limestone in the environment (Figure S3). It can be seen that the biosynthesis method for calcium carbonate has significant advantages_._ Additionally, BACC is a biologically sourced sustainable material, and its preparation process is highly straightforward, offering distinct advantages over alternative adsorbents. These characteristics underscore the exceptional performance and potential applications of BACC.

To elucidate the MB adsorption mechanism of BACC, we examined the temperature effect on its adsorption capacity. Table 1 shows a reduction in the *Q_m_* of BACC for MB as the temperature increased, indicating that the adsorption process is exothermic, potentially due to weakened physical forces and enhanced molecular movement at higher temperatures [33,36]. The value of Δ*G* increased with increasing temperatures in the temperature range of 283.15 to 328.15 K (Figure S4 and Table S2), showing a positive correlation (R^2^ > 0.96) and indicating that MB adsorption by BACC is thermodynamically unfavored [37]. The negative value of Δ*H* (−8.75 kJ·mol^−1^) verifies that adsorption between BACC and MB is an exothermic process [38,39], suggesting that an increase in temperature is detrimental to adsorption. Thus, it can be seen that the purification of dye wastewater by BACC will be favored in the winter months or in regions with cooler temperatures (e.g., northern China). The negative values of Δ*S* (−26.56 J/mol·K) revealed a decrease in randomness at the adsorbent–MB solution interface [40], indicating a more ordered transition state compared to the initial state. Similar adsorption phenomena were observed in the MB adsorption behaviors of activated carbon [32] defatted Carica papaya seeds [36], and walnut shell powder [41].

### 2.4. Adsorption Kinetics Analysis

The adsorption rate increased rapidly during the initial 5 min (Figure 2d) and maintained a plateau thereafter, indicating that equilibrium was reached with an adsorption capacity of 65.31 mg/g. This behavior arises from a decrease in the availability of adsorption sites on BACC, which restricts further interaction with MB. The PFO and PSO models were used to estimate kinetic parameters (Table S3) and examine kinetic data (Figure 2d). The PSO model, with a higher correlation coefficient (R^2^ = 0.9333) and a more consistent relationship between the experimental *Q_e_* value (65.31 mg/g) and the estimated *Q_e_*_2,*cal*_ (64.07 mg/g), provided a more accurate description of the adsorption process than the PFO model, which demonstrated that MB adsorption by BACC was controlled by chemisorption [5,42]. Furthermore, compared to other commonly used adsorption materials, BACC had a much lower *K*_2_ parameter (Table S4), indicating a faster rate of MB adsorption [43]. To delve deeper into the rate-determining step of the adsorption process, the kinetic behavior of BACC was examined using an ID model. The corresponding fitting outcomes are presented in the inset of Figure 2d and in Table S5. It is evident that the adsorption of MB by BACC proceeds through three distinct phases. The initial phase is characterized by a swift external diffusion, during which MB molecules migrate from the solution bulk to the BACC surface. The subsequent phase is indicative of progressive adsorption, suggesting that MB molecules commence their penetration into the pores of the adsorbent, with the adsorption rate being governed by intraparticle diffusion. The final phase is designated as the equilibrium phase, wherein MB molecules become adsorbed onto the internal surface area of the adsorbent. Furthermore, the simulated parameters derived from these calculations are detailed in Table S5. As indicated, the non-zero values of *C*_2_ suggest that the adsorption process of MB by BACC encompasses both intraparticle diffusion and external diffusion [44,45]. Accordingly, the adsorption process is simultaneously determined by multiple stages.

### 2.5. The Effect of pH

**Figure 3:**
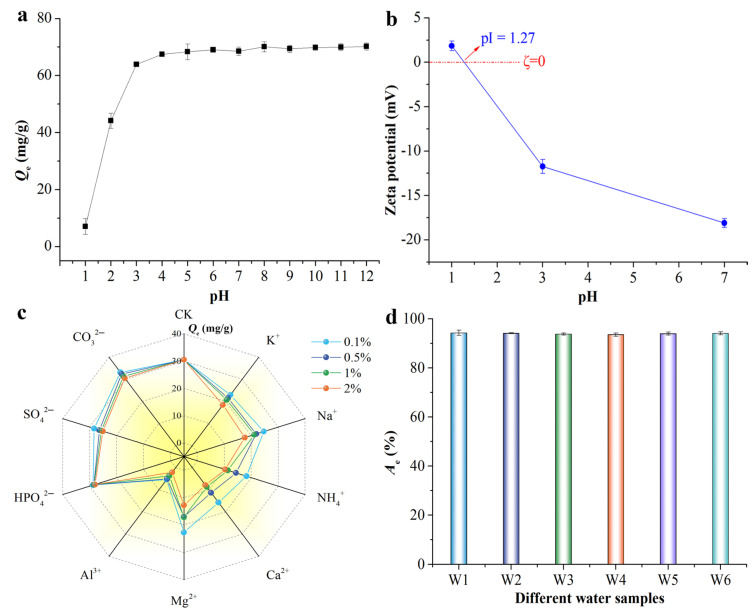
The Qe of BACC over a pH range of 1–12 (**a**), the zeta potential of BACC under various pH conditions (**b**), the Qe of MB by BACC with various coexisting substances (**c**), and the effect of different water samples on MB adsorption by BACC (**d**), W1: deionized water, W2: tap water, W3: lake water from Jinggangshan University, W4: Ganjiang River water, W5: lake water from Hangzhou West Lake, W6: real dyeing wastewater from a dyeing and finishing company in Zhejiang Province of China. Data represent the mean ± standard deviation (s.d.) from three independent experiments.

The adsorbent’s surface charge and the dye’s speciation distribution are both affected by the solution phase’s pH, which subsequently impacts dye adsorption [42,43]. Under pH conditions below 3, MB adsorption performance was found to be significantly lower (Figure 3a). Specifically, *Q_e_* was lowest at pH 1 due to the complete dissolution of BACC. The *Q_e_* rose considerably with increasing pH in the pH range of 1 to 3. After that, the adsorption capacity continued to be stable at about 67 mg/g and did not change considerably when the pH increased. As a result, BACC maintained a high adsorption capacity in both alkaline and acidic conditions (pH 3–12), demonstrating that it has an exceptional capacity to withstand H^+^ or OH^-^ ion interference, as it was almost unaffected by pH. The pH at which the zeta potential of BACC reaches zero was found to be around 1.27 (Figure 3b), which suggests the presence of negatively charged surfaces on the adsorbents [37]. Hence, the superior adsorptive properties of BACC can be attributed to the abundant negative charges on its surface, which engage in potent electrostatic interactions with MB across a diverse pH range.

### 2.6. The Effect of Coexisting Substances and Different Water Bodies

As shown in Figure 3c, the presence of most inorganic anions, namely SO_4_^2−^, CO_3_^2−^, and HPO_4_^2−^, had no significant effect on the adsorption of MB by BACC. However, the presence of inorganic cations, namely K^+^, Na^+^, NH_4_^+^, Ca^+^, Mg^2+^, and Al^3+^, resulted in significant inhibition of MB adsorption, because they had a high affinity with BACC and tended to occupy the adsorption sites for cationic dye MB [46]. Al^3+^, especially, has a stronger affinity for negatively charged BACC, thus exhibiting a greater inhibitory effect on MB adsorption (Figure 3c). The adsorption of MB by BACC is progressively hindered as the valence of concurrent cations in the solution increases. This occurs because MB, being a cationic dye, competes with these cations for adsorption sites on the BACC surface, leading to a suppression of MB adsorption due to the presence of these cations [46]. Moreover, we found that these different water bodies have almost no effect on the MB adsorption performance of BACC, which can effectively remove dyes from real wastewater with a removal efficiency of up to 94% (Figure 3d), demonstrating great potential for application in wastewater with low concentration dye, which indicates that BACC has good adsorption anti-interference performance.

### 2.7. Regeneration Experiment

**Figure 4:**
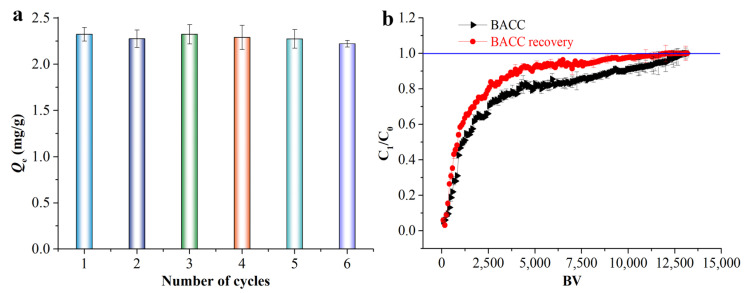
Regeneration experiment (**a**), and breakthrough curves of BACC and recovered BACC for MB in real dyeing wastewater (**b**), the blue line is the broken through point. Data represent the mean ± standard deviation (s.d.) from three independent experiments.

Regenerative capacity is a crucial parameter for evaluating the feasibility of adsorbents [47,48]. Figure 4a shows that after six sorption–desorption cycles, the MB *Q_e_* remained relatively unchanged, decreasing only slightly from 2.32 to 2.22 mg/g. Based on the breakthrough curve of MB from real dyeing wastewater in the fixed-bed column, BACC was found to be highly capable in the treatment of MB, with the whole fixed-bed column being broken through at about 13,000 BV (Figure 4b). The performance of the fixed-bed column was reduced from 13,000 to 12,000 BV after simple recovery using 50% ethanol washing, but the percentage of performance reduction was small (7.69%). Consequently, BACC has strong regeneration capabilities, featuring a simple regeneration process and low associated costs. In comparing BACC with and analyzing the existing treatment materials for dye wastewater in Zhejiang Province, BACC has great advantages in cost and efficiency.

### 2.8. Proposed Adsorption Mechanisms

**Figure 5:**
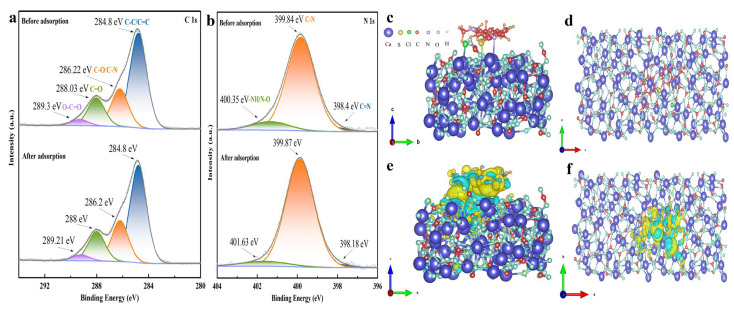
The XPS spectra of C1s (**a**) and N1s (**b**) before and after MB adsorption by BACC, the side (**c**) and top (**d**) views of the MB cation on the BACC surface, and differential charge density distributions for MB adsorbed on BACC (**e**,**f**), where the isosurface was adjusted to 0.001 electrons/Born3, and yellow and blue colors, respectively, represent gains and losses of electrons.

Biomineralization is an eco-friendly, energy-saving synthesis method that offers distinct advantages over traditional chemical synthesis in the laboratory, such as the unique properties of organic–inorganic hybrid materials, which can maintain the stability of the mesoporous structure, making them superior to common and synthetic minerals in the laboratory for achieving various important functions in living organisms or the environment [49,50,51]. The role of ACC in the environment is readily disregarded due to its thermodynamic instability, which usually results in its transformation into the more stable form of calcite [17,52]. Notably, there were no noticeable changes in the XRD patterns of BACC samples that had been stored as suspensions for three years before being heated at 100 °C or 200 °C for 2 h (Figure S5), indicating that the ACC made by bacteria (BACC) has great stability according to our new findings. As shown in Figure 1c, BACC comprises organic matter and bacterial cell debris; it can stabilize the ACC type and sustain its structure; and the organic functional groups it contains can establish hydrogen bonds with MB, improving MB adsorption. XPS analysis before and after the adsorption of MB by BACC showed slight shifts in the binding energies of C1s and N1s (Figure 5a,b), indicative of hydrogen bond formation [53,54]. The FTIR spectra of BACC before and after the adsorption of MB are shown in Figure S6. A decrease in the intensity of several absorption bands is observed, corresponding to the functional groups -OH, C=O, N-H, C-O, and -SH. This decrease is accompanied by a shift to lower wavelengths, which is attributed to the formation of hydrogen bonds and coordination compounds during the adsorption process [48,54,55]. Additionally, due to its exceptionally low isoelectric point (pI = 1.27), BACC exhibits a high degree of acid resistance and maintains a predominantly negative surface charge in aqueous solutions (Figure S7). This characteristic renders BACC particularly effective for the adsorption of cationic MB. Consequently, electrostatic interactions play a significant role in the MB adsorption process of BACC.

**Figure 6:**
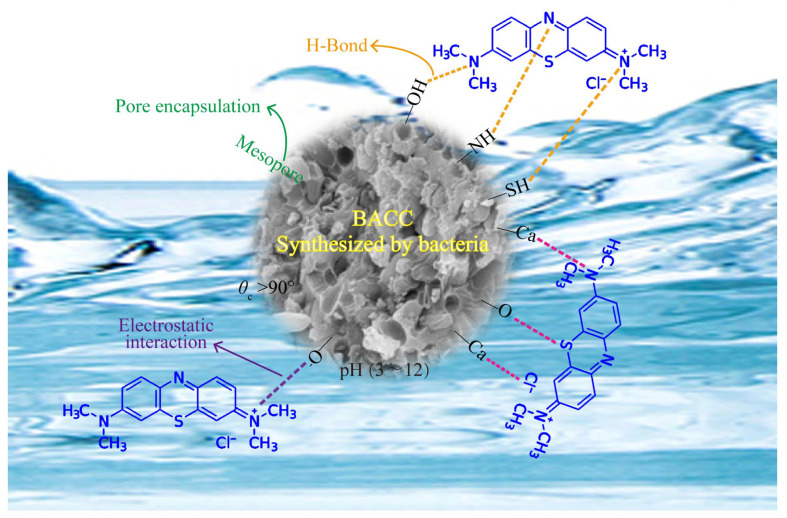
The possible mechanisms of efficient MB adsorption by BACC.

The pore structure and size distribution of BACC were examined. The N_2_ adsorption/desorption points on BACC’s adsorption isotherm show an adsorption hysteresis loop, with pore sizes primarily distributed between 1.8 and 3.5 nm (Figure S8a). BACC is classified as a typical mesoporous material by the IUPAC classification system because its N_2_ adsorption isotherm exhibits type IV characteristics of the H3 hysteresis loop (P/P^0^ > 0.4) [56,57]. Small-angle XRD analysis shows no diffraction peak (Figure S8b), confirming that the mesopores of BACC are disordered-mesoporous [49,58]. Therefore, these well-developed mesoporous configurations and a porous surface layer on BACC can encapsulate MB. The contact angle (*θ*_c_) of BACC is 133.69° (Figure S8c), suggesting that BACC has excellent hydrophobicity [59,60]; therefore, water molecules have minimal effect on the hydrophobic BACC when BACC adsorbs MB in water. In addition, we found that BACC was visible in water with an obvious Tyndall effect (Figure S8d), indicating that BACC has excellent colloidal properties and is well dispersed in water, thus facilitating the adsorption of MB in water. According to the results of density functional theory (DFT), MB forms Cl-Ca, S-O, and N-Ca bonds on the surface of BACC when it is adsorbed (Figure 5c,d), with an adsorption energy (*E_ads_*) of −4.65 eV. This negative *E_ads_* value indicates that the adsorption mechanism is exothermic [53,61], aligning with thermodynamic findings that higher temperatures lead to less adsorption of MB by BACC (Figure 2b and Table 1). The differential charge density results suggest that these electrons transfer from the BACC surface to MB (Figure 5e,f), which enhances the adsorption force [62]. Therefore, electrostatic attraction, along with Cl-Ca, S-O, N-Ca, and hydrogen bonds between MB and BACC, are the main forces driving adsorption (Figure 6). Overall, this study’s findings provide an innovative material with numerous benefits, marking a significant contribution to dye wastewater purification.

## 3. Materials and Methods

### 3.1. Preparation of BACC and Its Characterization

Bacterial seed liquid: In a liquid culture medium, two or three rings of *B. licheniformis* (CGMCC No. 24275) were inserted. The medium consisted of lysogeny broth (LB, 200 mL), with yeast extract (0.5% m/V), and 1% m/V of tryptone and NaCl, and had a pH of around 7.0. The bacterial seed liquid (with (3.85 ± 0.49) × 10^7^ cfu/mL concentration), was produced by shaking the mixture for around 12 h at 30 °C and 180 rpm.

Preparation of BACC: A total of 300 mL of LB liquid medium with CaCl_2_ (0.6 g) was poured into a sterile conical flask (500 mL). Following sterilization for 30 min at 115 °C, the BACC experimental group was established, cooled, and inoculated with bacterial seed liquid (5 mL). BACC synthesis was facilitated by agitating the experimental group consisting of 30 duplicates at 180 rpm after they were cultured at 30 °C for 7 days. After 7 days, each product was centrifuged for 15 min at 8000 rpm. The sediment was cleaned with double-distilled water, allowed to dry at 55 °C, and then milled using an agate mortar to a mesh size of 100 or less to prepare BACC products.

The BACC was examined using the following techniques: Fourier transform infrared spectrophotometry (FTIR, Nicolet iS50, Thermo Fisher, Waltham, MA, USA); energy-dispersive spectroscopy (EDS, Ultim Max65, Oxford, Abingdon, UK); AFM (Dimension Icon, Bruker, Billerica, MA, USA); an optical contact angle meter (OCA20, Dataphysics, Filderstadt, Germany); X-ray diffraction (XRD, BTXIII, Olympus, Shinjuku City, Japan); X-ray photoelectron spectroscopy (XPS, Escalab 250Xi, Thermo Fisher); and thermogravimetry–differential scanning calorimetry (TG-DSC, STA-449C, Netzsch, Burlington, MA, USA). The specific surface area of the samples was examined using a fully functional multi-purpose gas adsorption apparatus (3Flex, Micromeritics, Norcross, GA, USA). Before measurement, the samples were degassed at 100 °C for 24 h to avoid any potential structural damage to the adsorption material caused by high temperatures. The Barrett–Joyner–Halenda (BJH) method equation was employed to calculate the pore size distribution [49].

### 3.2. The Effects of Adsorbent Dose

To examine the influence of adsorbent dosage, a series of centrifuge tubes with MB (200 mg/L, 20 mL) solution (pH = 5.0) and BACC samples (0.005, 0.01, 0.03, 0.05, 0.07, and 0.1 g) were mixed and stirred for 24 h at 100 rpm and 25 °C. Using an ultraviolet–visible (UV-vis, λ_max_ = 664 nm) spectrophotometer (UV-2700i, Shimadzu, Kyoto, Japan) [5,53,63], the MB concentration in the supernatant was examined after centrifugation at 10,000 rpm for 10 min. Equations (1) and (2) were used to determine *Q_e_* (mg/g) and *A_e_* (%) [64].

(1) $$Q_{e}\;(\mathrm{mg}/g)=\frac{{(C}_{i}-C_{e})\times V}{W}$$

(2) $$A_{e}\;(\%)=\frac{C_{i}-C_{e}}{C_{i}}\times 100$$

where *C_i_* and *C_e_* are the starting and equilibrium MB concentrations in mg/L, *V* denotes the liquid volume at equilibrium contained in the centrifuge tube (L), and *W* denotes the BACC mass (g).

### 3.3. Adsorption Isotherms and Thermodynamic Analysis

MB stock solution was made with C_16_H_18_ClN_3_S·3H_2_O and diluted before use. MB solutions (pH = 5.0) were introduced to 50 mL centrifugal tubes containing 0.05 g BACC at different concentrations (10–4000 mg/L) until the volume reached 20 mL. At varying temperatures (10, 25, 40, 55 °C), all of the experimental batches were shaken for 24 h at 100 rpm.

After centrifugation for 10 min at 10,000 rpm, the supernatant was obtained. The quantity of MB was then determined through UV-Vis spectroscopy. The MB adsorption capacity (*Q_e_*) and adsorption efficiency (*A_e_*) of each adsorbent were calculated using the following expressions [63,65].

The experimental data were analyzed using the Freundlich and Langmuir adsorption models. The Langmuir equation was then used to calculate the MB’s maximum adsorption capacity (*Q_m_*) [54,66,67]:

(3) $$\mathrm{Langmuir}:\;Q_{e}=\frac{Q_{m}K_{L}C_{e}}{1+K_{L}C_{e}}$$

(4) $$\mathrm{Freundlich}:\;Q_{e}={K_{F}C_{e}}^{\frac{1}{n}}$$

The maximum adsorption capacity is denoted by *Q_m_*, while *K_L_* represents the Langmuir model’s coefficient (L/mg) in these equations. The variables 1/*n* and *K_F_* are the intensity and capacity factors of the Freundlich model, respectively.

The changes in enthalpy (Δ*H*), entropy (Δ*S*), and Gibbs free energy (Δ*G*) were calculated using the Van’t Hoff equation [68]:

(5) $$\ln K_{c}=\frac{\Delta S}{R}-\frac{\Delta H}{RT}$$

(6) $$\Delta G=-RT\ln K_{c}$$

where *K_c_* is the adsorption process’s thermodynamic equilibrium constant, *T* (K) is the absolute temperature [*T* (K) = *Temp.* (°C) + 273.15], and *R* denotes the molar gas constant (8.314 J·mol^−1^·K^−1^).

In addition, the same adsorption system was used to further investigate the differences in the MB adsorption capacity (100 mg/L) of BACC, of chemically synthesized calcium carbonate (Analytical pure calcium carbonate, APCC), and of limestone from the environment. Finally, the BACC composition was evaluated before and after MB was adsorbed, using XPS and FTIR. This analysis provided valuable information about the adsorption mechanisms employed by the BACC sample.

### 3.4. Analysis of Adsorption Kinetics

The adsorption kinetics analysis was conducted using the same procedure as the isotherm analysis. The BACC (0.05 g) and MB (200 mg/L, 20 mL; pH = 5.0) solutions in this case were incubated at 100 rpm and 25 °C for different lengths of time between 1 and 1400 min before being centrifuged (1 min, 15,000 rpm). The adsorption capacity at any time (min), denoted as *Q_t_*, can be calculated using the formula below:

(7) $$Q_{e}\;(\mathrm{mg}/g)=\frac{{(C}_{i}-C_{t})\times V}{W_{1}}$$

where *C_i_* and *C_t_* denote the initial MB concentrations in mg/L at time *t*, respectively, *V* represents the MB solution’s volume (L), and *W*_1_ denotes the adsorbent’s mass in g.

Adsorption kinetics may provide very beneficial information on the characteristics of the adsorption mechanism [69]. Adsorption data are analyzed by nonlinearized kinetic models, including the pseudo-second-order (PSO), pseudo-first-order (PFO), and interparticle diffusion (ID) models, which are represented mathematically as follows [45,69]:

(8) $$Q_{t}=Q_{e}(1-e^{-K_{1}t})$$

(9) $$Q_{t}=\frac{K_{2}{Q_{e}}^{2}t}{1+K_{2}Q_{e}t}$$

(10) $$Q_{t}={K_{id}t}^{\frac{1}{2}}+C$$

Here, *Q_e_* and *Q_t_* (mg/g) are, respectively, the MB adsorption capacity at equilibrium and at any time (min); *K*_1_ (min^−1^), *K*_2_ (g·(mg·min)^−1^), and *K_id_* (mg·(g·min^1/2^)^−1^) represent, respectively, the rate constant of the PFO, PSO and ID models; and C (mg·g^−1^) is a constant related to the thickness of the boundary layer in the ID model.

### 3.5. The Alterations Caused by pH

To perform the adsorption analysis, BACC samples (0.05 g) were put in a set of centrifugal tubes containing MB (200 mg/L, 20 mL) solution, with the initial pH of the tubes adjusted between 1 and 12. The samples were prepared in triplicate and subjected to agitation at 25 °C and 100 rpm for 24 h. Following the adsorption process, the reaction products were centrifuged for 10 min at 10,000 rpm. A UV-vis spectrophotometer was then used to measure the concentrations of MB in the supernatants, and Equation (1) was used to calculate the *Q_e_* for MB. Furthermore, the zeta potential of BACC was evaluated under a variety of pH conditions (1, 3, 7).

### 3.6. The Effect of Coexisting Ions

The impact of various coexisting ions on the adsorption performance of BACC was investigated to assess its application potential in more detail. The BACC samples (0.05 g) were placed in a series of centrifuge tubes with various coexisting ions K^+^ (0–2%), Na^+^ (0–2%), NH_4_^+^ (0–2%), Ca^2+^ (0–2%), Mg^2+^ (0–2%), Al^3+^ (0–2%), SO_4_^2−^ (0–2%), CO_3_^2−^ (0–2%), and HPO_4_^2−^ (0–2%). Each tube contained a 20 mL solution of MB (100 mg/L), and the mixture was agitated at 100 rpm and 25 °C for 24 h. MB concentration in the supernatant was examined using a UV-vis spectrophotometer following centrifugation at 10,000 rpm for 10 min. *Q_e_* (mg/g) was determined utilizing Equation (1).

### 3.7. The Effect of Different Water Bodies

The BACC samples (0.05 g) were placed in a series of centrifuge tubes containing water samples from various water bodies, such as deionized water, tap water, lake water from Gardener Lake in Jinggangshan University, river water from Ganjiang River, lake water from Hangzhou West Lake, and real dyeing wastewater from a dyeing and finishing company in the Zhejiang Province of China. Each tube contained a 20 mL solution of MB (10 mg/L, in reference to the actual concentration of wastewater), and the mixture was agitated at 100 rpm and 25 °C for 24 h. MB concentration in the supernatant was examined using a UV-vis spectrophotometer following centrifugation at 10,000 rpm for 10 min. *A_e_* (%) was determined utilizing Equation (2).

### 3.8. Regeneration Experiment and Practical Application

The adsorption experiments utilized regeneration to test pollutants for the desorption regeneration experiments. BACC (0.05 g) was added to real dyeing wastewater consisting of MB (20 mL, 10 mg/L; the real dyeing wastewater came from a dyeing and finishing company in the Zhejiang Province of China) and agitated for 24 h at 25 °C and 100 rpm. The mixture was centrifuged to prepare for the subsequent adsorption experiment. The precipitate was then regenerated by immersing it in 20% anhydrous ethanol and agitating it at 100 rpm and 25 °C for 24 h [70]. To determine *Q_e_* (mg/g), the amounts of each pollutant in the supernatant were measured. This adsorption/desorption experimental cycle was repeated six times. In addition, a fixed-bed apparatus (Figure S1) was further used to plot the breakthrough curves of BACC and MB adsorbed by BACC after 50% ethanol elution regeneration [71], based on the ratio of solution volume to adsorbent volume (BV) and residual rate (*C*_1_/*C*_0_, where *C*_1_ is the concentration of the adsorption equilibrium solution and *C*_0_ is the initial concentration of 10 mg/L), to evaluate their potential for regeneration applications.

### 3.9. DFT-Based Theoretical Evaluation of Interatomic Adsorption

The Vienna Ab-initio Simulation Package (VASP, v6.3.2) [72] was employed to execute structural optimization using the projector augmented wave (PAW) method [73]. The weak interactions between atoms were described by treating the exchange functional with the Perdew–Burke–Ernzerhof (PBE) functional [74] and the DFT-D3 correction [75]. During structural optimization, the cut-off energy value for the plane-wave basis was determined to be 450 eV. Brillouin zone integration was carried out using a 0.03 Å^−1^ Monkhorst-Pack *k*-point mesh for geometry and lattice size optimization. The Kohn–Sham orbitals were permitted to have partial occupancies with widths of 0.05 eV using the Gaussian smearing approach. For geometric optimization, a change in energy of less than 0.02 eV Å^−1^ was considered convergent. At the Γ-point, integration in reciprocal space was implemented in first-principles molecular dynamic (MD) calculations. The MD calculations were conducted with a time step of 2 fs. With a Nosé thermostat set to 3000 K for 10 ps, an equilibration step was completed in the canonical ensemble (constant N, V, and T). After that, the temperature was annealed to room temperature. The BACC surface was modeled with a 15 Å vacuum in the z direction to separate the slabs. The charge density distribution isosurface was calculated by the optB88-vdW functional [76]. The formula below was used to calculate adsorption energy (*E_ads_*) [33,54,77]:

*E*_*ads*_ = *E*_*total*_ − *E*_*MB*_ − *E*_*sub*_ (11)

where *E_total_* is the total energy of adsorption, *E_MB_* is the energy of the free adsorbate molecule in the gas phase, and *E_su__b_* is the energy of its clear surface. *E_ads_* numbers in the negative signify exothermic adsorption, while higher negative values denote stronger adsorption [61].

## 4. Conclusions

The widespread presence of calcium carbonate has a major impact on the transformation or movement of dyes in different aquatic environments. Our observations revealed a novel BACC adsorbent that is low-cost, eco-friendly, recyclable, and can be facilely synthesized. The favorable adsorption characteristics of BACC for MB are attributed to its hydrophobic property, porosity, electronegativity, low energy barrier, and perfect dispersity in aqueous solution. Cl-Ca, S-O, N-Ca, H-bonding, electrostatic attraction, and pore encapsulation are the main driving forces of adsorption between MB and BACC. Moreover, BACC maintained a relatively strong adsorption capacity under various environmental conditions (temperatures, pH, coexisting ions, and different water bodies). This study presents a highly efficient capture material for cationic dye with practical applications in real environmental scenarios, offering valuable insights and technical solutions for the treatment of dye-contaminated water bodies.

## Figures and Tables

**Table 1:** Isotherm coefficients as per Langmuir and Freundlich models.

*T* (°C)	Langmuir				Freundlich			
	*Q_m_* (mg/g)	*K_L_* (L/mg)	R^2^	ARE	1/*n*	*K*_F_ (mg^1−(1/*n*)^ · L^1/*n*^/g)	R^2^	ARE
10	544.87	0.006	0.9921	0.9914	0.35	34.168	0.8939	0.8842
25	494.86	0.005	0.9885	0.9875	0.36	28.367	0.8925	0.8827
40	470.86	0.003	0.9896	0.9887	0.40	18.513	0.9120	0.9040
55	414.55	0.003	0.9938	0.9933	0.42	13.884	0.9272	0.9206

## Data Availability

Data are contained within the article and Supplementary Materials.

## Supplementary Materials

The following supporting information can be downloaded at: https://www.mdpi.com/article/10.3390/molecules29225426/s1, Figure S1: Fixed-bed device (MB: 10 mg/L, flow rate 3 mL/min, filter column (10 mL) filled with 0.1 mL BACC and 9.9 mL quartz fine sand, outlet water sample collection time interval of 3 min). Figure S2: Before- and after-MB adsorption color changes of the BACC sample. Figure S3: The MB adsorption capacity of BACC, chemically synthesized calcium carbonate (Analytical pure calcium carbonate, APCC), and limestone from the environment. (Bars with different letters indicate statistically significant differences, one-way ANOVA, Duncan’s multiple range test, *p* < 0.01.) Figure S4: Δ*G* versus temperature for MB adsorption by BACC. Figure S5: XRD patterns of BACC in water for 3 years and heated at 100 °C, or at 200 °C for 2 h. Figure S6. FTIR results of BACC before and after MB adsorption. Figure S7: Involvement of electrostatic interaction in MB adsorption by BACC. (ΔpH = pH_e_ − pH_0_, pH_e_ is the equilibrium pH value; pH_0_ is the initial pH value.) Figure S8: The N_2_-sorption isotherms and the pore size distribution (inset) (a), small-angle XRD pattern (b), contact angle (c), and Tyndall phenomenon (d) of tested BACC. Table S1: A comparison of the *Q_m_* values obtained for MB. Table S2: Thermodynamic parameters for MB adsorption by BACC. Table S3: Kinetic constants of the PFO and PSO models for MB adsorption by BACC. Table S4: Comparison of the *K*_2_ values of a PSO kinetic model for MB. Table S5. Fitting parameters of the ID model for adsorption of MB by BACC. References [34,35,36,78,79,80,81,82,83,84,85,86,87,88,89,90,91,92,93,94,95,96] are cited in the Supplementary Materials.

## References

[1] Chen H., Liu Y., Xu X., Sun M., Jiang M., Xue G., Li X., Liu Z. (2019). How does iron facilitate the aerated biofilter for tertiary simultaneous nutrient and refractory organics removal from real dyeing wastewater?. Water Res..

[2] Walter A.D., Benamor H., Ferrer L.M., Reji T., Curran T., Schwenk G.R., Hadji M., Creighton M.A., Barsoum M.W. (2024). Self-sensitized photodegradation and adsorption of aqueous malachite green dye using one-dimensional titanium oxide nanofilaments. iScience.

[3] Mujtaba G., Ullah A., Khattak D., Shah M.U.H., Daud M., Ahmad S., Hai A., Ahmed F., Alshahrani T., Banat F. (2023). Simultaneous adsorption of methylene blue and amoxicillin by starch-impregnated mgal layered double hydroxide: Parametric optimization, isothermal studies and thermo-kinetic analysis. Environ. Res..

[4] Obayomi K.S., Lau S.Y., Danquah M.K., Zhang J., Chiong T., Meunier L., Gray S.R., Rahman M.M. (2023). Green synthesis of graphene-oxide based nanocomposites for efficient removal of methylene blue dye from wastewater. Desalination.

[5] Pervez M.N., Hassan M.M., Naddeo V. (2024). Separation of cationic methylene blue dye from its aqueous solution by s-sulfonated wool keratin-based sustainable electrospun nanofibrous membrane biosorbent. Sep. Purif. Technol..

[6] Shi T., Yang B., Hu W., Gao G., Jiang X., Yu J. (2024). Garlic peel-based biochar prepared under weak carbonation conditions for efficient removal of methylene blue from wastewater. Molecules.

[7] Mondal M.I.H., Chandra Chakraborty S., Rahman M.S., Marjuban S.M.H., Ahmed F., Zhou J.L., Ahmed M.B., Zargar M. (2024). Adsorbents from rice husk and shrimp shell for effective removal of heavy metals and reactive dyes in water. Environ. Pollut..

[8] Teo S.H., Ng C.H., Islam A., Abdulkareem-Alsultan G., Joseph C.G., Janaun J., Taufiq-Yap Y.H., Khandaker S., Islam G.J., Znad H. (2022). Sustainable toxic dyes removal with advanced materials for clean water production: A comprehensive review. J. Clean. Prod..

[9] Flilissa A., Laouameur K., Hammoudi N., Tamam N., Yadav K.K., Achouri B., Alyami A.Y., Flilissa O., Algethami J.S., Abbas M. (2024). Bentonite sdbs-loaded composite for methylene blue removal from wastewater: An experimental and theoretical investigation. Environ. Res..

[10] Wolski R., Bazan-Wozniak A., Nosal-Wiercińska A., Pietrzak R. (2024). Methylene blue and rhodamine b dyes’ efficient removal using biocarbons developed from waste. Molecules.

[11] Pan Y., Wang Y., Zhou A., Wang A., Zhu T. (2017). Removal of azo dye in an up-flow membrane-less bioelectrochemical system integrated with bio-contact oxidation reactor. Chem. Eng. J..

[12] Liu R., Huang S., Zhang X., Song Y., He G., Wang Z., Lian B. (2021). Bio-mineralisation, characterization, and stability of calcium carbonate containing organic matter. RSC Adv..

[13] Tang S., Chang X., Li M., Ge T., Niu S., Wang D., Jiang Y., Sun S. (2021). Fabrication of calcium carbonate coated-stainless steel mesh for efficient oil-water separation via bacterially induced biomineralization technique. Chem. Eng. J..

[14] Mehta N., Vantelon D., Gaëtan J., Fernandez-Martinez A., Delbes L., Travert C., Benzerara K. (2023). Calcium speciation and coordination environment in intracellular amorphous calcium carbonate (acc) formed by cyanobacteria. Chem. Geol..

[15] Dhami N.K., Reddy M.S., Mukherjee A. (2013). Biomineralization of calcium carbonates and their engineered applications: A review. Front. Microbiol..

[16] Giuseppe F., Simona F., Michela R., Branka N.D.A., Damir K. (2014). Evidence of structural variability among synthetic and biogenic vaterite. Chem. Commun..

[17] Zou Z., Yang X., Albéric M., Heil T., Wang Q., Pokroy B., Politi Y., Bertinetti L. (2020). Additives control the stability of amorphous calcium carbonate via two different mechanisms: Surface adsorption versus bulk incorporation. Adv. Funct. Mater..

[18] Dupraz C., Reid R.P., Braissant O., Decho A.W., Norman R.S., Visscher P.T. (2009). Processes of carbonate precipitation in modern microbial mats. Earth-Sci. Rev..

[19] Liu R., Guan Y., Chen L., Lian B. (2018). Adsorption and desorption characteristics of Cd^2+^ and Pb^2+^ by micro and nano-sized biogenic CaCO_3_. Front. Microbiol..

[20] Liu R., Yu Y., Liu X., Guan Y., Chen L., Lian B. (2018). Adsorption of Ni^2+^ and Cu^2+^ using bio-mineral: Adsorption isotherms and mechanisms. Geomicrobiol. J..

[21] Kim J., Won Y., Ji C., Yang Y., Ryu S., Ju S., Kwon Y., Lee Y., Lee J. (2017). The difference in in vivo sensitivity between *Bacillus licheniformis* perr and *Bacillus subtilis* perr is due to the different cellular environments. Biochem. Biophys. Res. Commun..

[22] Pan J., Wang G., Nong J., Xie Q. (2023). Biodegradation of benzo(a)pyrene by a genetically engineered *Bacillus licheniformis*: Degradation, metabolic pathway and toxicity analysis. Chem. Eng. J..

[23] Enyedi N.T., Makk J., Kótai L., Berényi B., Klébert S., Sebestyén Z., Molnár Z., Borsodi A.K., Leél-Qssy S., Demény A. (2020). Cave bacteria-induced amorphous calcium carbonate formation. Sci. Rep..

[24] Rodriguez-Navarro C., Kudłacz K., Cizer Ö., Ruiz-Agudo E. (2015). Formation of amorphous calcium carbonate and its transformation into mesostructured calcite. CrystEngComm.

[25] Martignier A., Pacton M., Filella M., Jaquet J.M., Barja F., Pollok K., Langenhorst F., Lavigne S., Guagliardo P., Kilburn M.R. (2017). Intracellular amorphous carbonates uncover a new biomineralization process in eukaryotes. Geobiology.

[26] Li H., Yao Q., Wang F., Huang Y., Fu S., Zhou G. (2019). Insights into the formation mechanism of vaterite mediated by a deep-sea bacterium *Shewanella piezotolerans* wp3. Geochim. Cosmochim. Acta.

[27] Wu Z., Zhong H., Yuan X., Wang H., Wang L., Chen X., Zeng G., Wu Y. (2014). Adsorptive removal of methylene blue by rhamnolipid-functionalized graphene oxide from wastewater. Water Res..

[28] Rodriguez-Navarro C., Jimenez-Lopez C., Rodriguez-Navarro A., Gonzalez-Munoz M.T., Rodriguez-Gallego M. (2007). Bacterially mediated mineralization of vaterite. Geochim. Cosmochim. Acta.

[29] Lyu J., Li F., Zhang C., Gower L., Wasman S., Sun J., Yang G., Chen J., Gu L., Tang X. (2021). From the inside out: Elemental compositions and mineral phases provide insights into bacterial calcification. Chem. Geol..

[30] Wu Q., Siddique M.S., Wu M., Wang H., Zhang Y., Yang R., Cui L., Ma W., Yan J., Yang Y. (2024). Synergistically enhancing the selective adsorption of cationic dyes through copper impregnation and amino functionality into iron-based metal-organic frameworks. Sci. Total Environ..

[31] Shukla A., Zhang Y.H., Dubey P., Margrave J.L., Shukla S.S. (2002). The role of sawdust in the removal of unwanted materials from water. J. Hazard. Mater..

[32] Aroguz A.Z., Gulen J., Evers R.H. (2008). Adsorption of methylene blue from aqueous solution on pyrolyzed petrified sediment. Bioresour. Technol..

[33] Luo J., Luo X., Crittenden J., Qu J., Bai Y., Peng Y., Li J. (2015). Removal of antimonite (sb(iii)) and antimonate (sb(v)) from aqueous solution using carbon nanofibers that are decorated with zirconium oxide (ZrO_2_). Environ. Sci. Technol..

[34] Heidarinejad Z., Rahmanian O., Fazlzadeh M., Heidari M. (2018). Enhancement of methylene blue adsorption onto activated carbon prepared from date press cake by low frequency ultrasound. J. Mol. Liq..

[35] Heidarizad M., Şengör S.S. (2016). Synthesis of graphene oxide/magnesium oxide nanocomposites with high-rate adsorption of methylene blue. J. Mol. Liq..

[36] Unuabonah E.I., Adie G.U., Onah L.O., Adeyemi O.G. (2009). Multistage optimization of the adsorption of methylene blue dye onto defatted carica papaya seeds. Chem. Eng. J..

[37] He C., Lin H., Dai L., Qiu R., Tang Y., Wang Y., Duan P., Ok Y.S. (2020). Waste shrimp shell-derived hydrochar as an emergent material for methyl orange removal in aqueous solutions. Environ. Int..

[38] Jain M., Khan S.A., Sahoo A., Dubey P., Pant K.K., Ziora Z.M., Blaskovich M.A.T. (2022). Statistical evaluation of cow-dung derived activated biochar for phenol adsorption: Adsorption isotherms, kinetics, and thermodynamic studies. Bioresour. Technol..

[39] Mani D., Elango D., Priyadharsan A., Al-Humaid L.A., Al-Dahmash N.D., Ragupathy S., Jayanthi P., Ahn Y. (2023). Groundnut shell chemically treated with koh to prepare inexpensive activated carbon: Methylene blue adsorption and equilibrium isotherm studies. Environ. Res..

[40] Egbosiuba T.C., Abdulkareem A.S., Kovo A.S., Afolabi E.A., Tijani J.O., Auta M., Roos W.D. (2020). Ultrasonic enhanced adsorption of methylene blue onto the optimized surface area of activated carbon: Adsorption isotherm, kinetics and thermodynamics. Chem. Eng. Res. Des..

[41] Miyah Y., Lahrichi A., Idrissi M., Khalil A., Zerrouq F. (2018). Adsorption of methylene blue dye from aqueous solutions onto walnut shells powder: Equilibrium and kinetic studies. Surf. Interfaces.

[42] Liu Y., Wang S., Huo J., Zhang X., Wen H., Zhang D., Zhao Y., Kang D., Guo W., Ngo H.H. (2024). Adsorption recovery of phosphorus in contaminated water by calcium modified biochar derived from spent coffee grounds. Sci. Total Environ..

[43] Li G., Zhu W., Zhang C., Zhang S., Liu L., Zhu L., Zhao W. (2016). Effect of a magnetic field on the adsorptive removal of methylene blue onto wheat straw biochar. Bioresour. Technol..

[44] Zhao R., Li X., Sun B., Li Y., Li Y., Yang R., Wang C. (2017). Branched polyethylenimine grafted electrospun polyacrylonitrile fiber membrane: A novel and effective adsorbent for cr(vi) remediation in wastewater. J. Mater. Chem. A.

[45] Yu Z., Wu Z., Sheng R., Liu C., Chen H., Zhang J., Qiu Z. (2023). Ultra-high adsorption of cr from aqueous solution using ldhs decorated magnetic hydrochar: Selectivity and anti-interference exploration. Sep. Purif. Technol..

[46] Su X., Wang X., Ge Z., Bao Z., Lin L., Chen Y., Dai W., Sun Y., Yuan H., Yang W. (2024). Koh-activated biochar and chitosan composites for efficient adsorption of industrial dye pollutants. Chem. Eng. J..

[47] Chen X., Yang M., Zheng S., Temprano-Coleto F., Dong Q., Cheng G., Yao N., Stone H.A., Hu L., Ren Z.J. (2023). Spatially separated crystallization for selective lithium extraction from saline water. Nat. Water.

[48] Li D., Sun L., Yang L., Liu J., Shi L., Zhuo L., Ye T., Wang S. (2024). Adsorption behavior and mechanism of modified pinus massoniana pollen microcarriers for extremely efficient and rapid adsorption of cationic methylene blue dye. J. Hazard. Mater..

[49] Liu R., Lian B. (2019). Immobilisation of cd(ii) on biogenic and abiotic calcium carbonate. J. Hazard. Mater..

[50] Espinosa H.D., Rim J.E., Barthelat F., Buehler M.J. (2009). Merger of structure and material in nacre and bone—Perspectives on de novo biomimetic materials. Prog. Mater. Sci..

[51] Wendler J.E., Bown P. (2013). Exceptionally well-preserved cretaceous microfossils reveal new biomineralization styles. Nat. Commun..

[52] Vecht A., Ireland T.G. (2000). The role of vaterite and aragonite in the formation of pseudo-biogenic carbonate structures: Implications for martian exobiology. Geochim. Cosmochim. Acta.

[53] Liu R., Zhang J., Fu H., Yin L., Song Y., He G. (2023). A comparative study of methylene blue adsorption and removal mechanisms by calcium carbonate from different sources. Bioresour. Technol..

[54] Duan R., Li W., Chen D., Cui T., Xiang T., Zhang Y., Wang H., Xu R. (2025). Co-removal of mercury and organic dye via the rectangle-shaped nanosheets loaded hydrochar: Surface interactions and dft calculations. Sep. Purif. Technol..

[55] Diao Z., Zhang L., Li Q., Gao X., Gao X., Seliem M.K., Dhaoudi F., Sellaoui L., Deng S., Bonilla-Petriciolet A. (2024). Adsorption of food dyes from aqueous solution on a sweet potato residue-derived carbonaceous adsorbent: Analytical interpretation of adsorption mechanisms via adsorbent characterization and statistical physics modeling. Chem. Eng. J..

[56] Wang X., Yang L., Zhang J., Wang C., Li Q. (2014). Preparation and characterization of chitosan-poly(vinyl alcohol)/bentonite nanocomposites for adsorption of hg(ii) ions. Chem. Eng. J..

[57] Zhao J., Chen J., Xu S., Shao M., Zhang Q., Wei F., Ma J., Wei M., Evans D.G., Xue D. (2014). Hierarchical nimn layered double hydroxide/carbon nanotubes architecture with superb energy density for flexible supercapacitors. Adv. Funct. Mater..

[58] Liu R., Shi Y., Wan Y., Meng Y., Zhang F., Gu D., Chen Z., Tu B., Zhao D. (2006). Triconstituent co-assembly to ordered mesostructured polymer-silica and carbon-silica nanocomposites and large-pore mesoporous carbons with high surface areas. J. Am. Chem. Soc..

[59] Wang Z., Yuan L., Liang G., Gu A. (2021). Mechanically durable and self-healing super-hydrophobic coating with hierarchically structured kh570 modified sio_2_-decorated aligned carbon nanotube bundles. Chem. Eng. J..

[60] Zhang Y., Zhu X., Chen B. (2020). Nanoscale profiling of 2d surface hydrophobicity recognition of environmental media via afm measurements in situ. Environ. Sci. Technol..

[61] Ofili N.E.R., Thetford A., Kaltsoyannis N. (2020). Adsorption of u(vi) on stoichiometric and oxidised mackinawite: A dft study. Environ. Sci. Technol..

[62] Meng R., Deng Q., Peng C., Chen B., Liao K., Li L., Yang Z., Yang D., Zheng L., Zhang C. (2020). Two-dimensional organic-inorganic heterostructures of in situ-grown layered cof on ti_3_c_2_ mxene nanosheets for lithium-sulfur batteries. Nano Today.

[63] Zong M., Song D., Zhang X., Huang X., Lu X., Rosso K.M. (2021). Facet-dependent photodegradation of methylene blue by hematite nanoplates in visible light. Environ. Sci. Technol..

[64] Ullah F., Ji G., Irfan M., Gao Y., Shafiq F., Sun Y., Ain Q.U., Li A. (2022). Adsorption performance and mechanism of cationic and anionic dyes by koh activated biochar derived from medical waste pyrolysis. Environ. Pollut..

[65] Minisy I.M., Salahuddin N.A., Ayad M.M. (2021). Adsorption of methylene blue onto chitosan–montmorillonite/polyaniline nanocomposite. Appl. Clay Sci..

[66] Langmuir I. (1918). The adsorption of gases on plane surfaces of glass, mica and platinum. J. Am. Chem. Soc..

[67] Wu M., Zhang Y., Feng X., Yan F., Li Q., Cui Q., Li B. (2023). Fabrication of cationic cellulose nanofibrils/sodium alginate beads for congo red removal. iScience.

[68] Dutta S., Srivastava S.K., Gupta B., Gupta A.K. (2021). Hollow polyaniline microsphere/mno_2_/fe_3_o_4_ nanocomposites in adsorptive removal of toxic dyes from contaminated water. ACS Appl. Mater. Interfaces.

[69] Tran H.N., You S., Hosseini-Bandegharaei A., Chao H. (2017). Mistakes and inconsistencies regarding adsorption of contaminants from aqueous solutions: A critical review. Water Res..

[70] Boukarma L., Aziam R., Aboussabek A., El Qdhy S., Zerbet M., Sinan F., Chiban M. (2024). Novel insights into crystal violet dye adsorption onto various macroalgae: Comparative study, recyclability and overview of chromium (vi) removal. Bioresour. Technol..

[71] Wang D., Song J., Wen J., Yuan Y., Liu Z., Lin S., Wang H., Wang H., Zhao S., Zhao X. (2018). Significantly enhanced uranium extraction from seawater with mass produced fully amidoximated nanofiber adsorbent. Adv. Energy Mater..

[72] Hafner J. (2008). Ab-initio simulations of materials using vasp: Density-functional theory and beyond. J. Comput. Chem..

[73] Blöchl P.E. (1994). Projector augmented-wave method. Phys. Rev. B.

[74] Burke K., Ernzerhof M., Perdew J.P. (1996). Generalized gradient approximation made simple. Phys. Rev. Lett..

[75] Grimme S. (2006). Semiempirical gga-type density functional constructed with a long-range dispersion correction. J. Comput. Chem..

[76] Hensley A.J.R., Wang Y., Mcewen J. (2016). Adsorption of guaiacol on fe (110) and pd (111) from first principles. Surf. Sci..

[77] Li S., Huang L., Zhang H., Huang Z., Jia Q., Zhang S. (2021). Adsorption mechanism of methylene blue on oxygen-containing functional groups modified graphitic carbon spheres: Experiment and dft study. Appl. Surf. Sci..

[78] Mouni L., Belkhiri L., Bollinger J., Bouzaza A., Assadi A., Tirri A., Dahmoune F., Madani K., Remini H. (2018). Removal of methylene blue from aqueous solutions by adsorption on kaolin: Kinetic and equilibrium studies. Appl. Clay Sci..

[79] Bilgi C. (2005). Investigation of the factors affecting organic cation adsorption on some silicate minerals. J. Colloid Interface Sci..

[80] Gürses A., Karaca S., Doğar Ç., Bayrak R., Açıkyıldız M., Yalçın M. (2004). Determination of adsorptive properties of clay/water system: Methylene blue sorption. J. Colloid. Interface. Sci..

[81] Su H., Guo X., Zhang X., Zhang Q., Huang D., Lin L., Qiang X. (2022). Ultrafine biosorbent from waste oyster shell: A comparative study of congo red and methylene blue adsorption. Bioresour. Technol. Rep..

[82] Albadarin A.B., Collins M.N., Mu N., Shirazian S., Mangwandi C. (2017). Activated lignin-chitosan extruded blends for efficient adsorption of methylene blue. Chem. Eng. J..

[83] Han R., Wang Y., Han P., Shi J., Yang J., Lu Y. (2006). Removal of methylene blue from aqueous solution by chaff in batch mode. J. Hazard. Mater..

[84] Lonappan L., Rouissi T., Das R.K., Brar S.K., Ramirez A.A., Verma M., Surampalli R.Y., Valero J.R. (2016). Adsorption of methylene blue on biochar microparticles derived from different waste materials. Waste Manag..

[85] Yan H., Tao X., Yang Z., Li K., Yang H., Li A., Cheng R. (2014). Effects of the oxidation degree of graphene oxide on the adsorption of methylene blue. J. Hazard. Mater..

[86] Peydayesh M., Rahbar-Kelishami A. (2015). Adsorption of methylene blue onto platanus orientalis leaf powder: Kinetic, equilibrium and thermodynamic studies. J. Ind. Eng. Chem..

[87] Yao Y., Xu F., Chen M., Xu Z., Zhu Z. (2010). Adsorption behavior of methylene blue on carbon nanotubes. Bioresour. Technol..

[88] Othman N.H., Alias N.H., Shahruddin M.Z., Abu Bakar N.F., Nik Him N.R., Lau W.J. (2018). Adsorption kinetics of methylene blue dyes onto magnetic graphene oxide. J. Environ. Chem. Eng..

[89] Özer D., Dursun G., Özer A. (2007). Methylene blue adsorption from aqueous solution by dehydrated peanut hull. J. Hazard. Mater..

[90] Gupta N., Kushwaha A.K., Chattopadhyaya M.C. (2016). Application of potato (solanum tuberosum) plant wastes for the removal of methylene blue and malachite green dye from aqueous solution. Arab. J. Chem..

[91] Zhao C., Luan J., Zhai Q., Liu W., Ge H., Ke X., Yan Z. (2022). Releasing sio tetrahedron and alo octahedron from montmorillonite to enhance the adsorption performance of carbon@chitosan@montmorillonite nanosheet for cationic dyes: Coupling quantum chemistry simulations with experiments. Sci. Total Environ..

[92] Lu K., Wang T., Zhai L., Wu W., Dong S., Gao S., Mao L. (2019). Adsorption behavior and mechanism of fe-mn binary oxide nanoparticles: Adsorption of methylene blue. J. Colloid. Interface. Sci..

[93] Niu J., Luo L., Cui J., Zhang H., Guo Y., Li L., Cheng F. (2023). Impact of inherent calcium in coal on the structure and performance of activated carbon in flue gas activation: The enhanced mechanism of calcite on the methylene blue adsorption. J. Clean. Prod..

[94] Rida K., Bouraoui S., Hadnine S. (2013). Adsorption of methylene blue from aqueous solution by kaolin and zeolite. Appl. Clay Sci..

[95] Vasiraja N., Saravana Sathiya Prabhahar R., Joshua A. (2023). Preparation and physio–chemical characterisation of activated carbon derived from prosopis juliflora stem for the removal of methylene blue dye and heavy metal containing textile industry effluent. J. Clean. Prod..

[96] Yao X., Ji L., Guo J., Ge S., Lu W., Cai L., Wang Y., Song W., Zhang H. (2020). Magnetic activated biochar nanocomposites derived from wakame and its application in methylene blue adsorption. Bioresour. Technol..

