# Supplementary material for: Efficient Removal of Cationic Dye by Biomimetic Amorphous Calcium Carbonate: Behavior and Mechanisms
Source: Molecules. 2024 Nov 18;29(22):5426. doi: 10.3390/molecules29225426 (PMC11597820; doi:10.3390/molecules29225426)
Supplement: Supplementary file 1 [file molecules-29-05426-s001.zip › molecules-3288745-supplementary.pdf]

Supplementary Material

Efficient Removal of Cationic Dye by Biomimetic Amorphous Calcium Carbonate: Behavior and Mechanisms

Renlu Liu, Weizhen Ji, Jie Min, Pengjun Wen, Yan Li, Jialu Hu, Li Yin, Genhe He *

Key Laboratory of Jiangxi Province for Functional Biology and Pollution Control in Red Soil Regions, School of Life Sciences, Jinggangshan University, Ji'an 343009, China

* Correspondence: hegenhe@jgsu.edu.cn

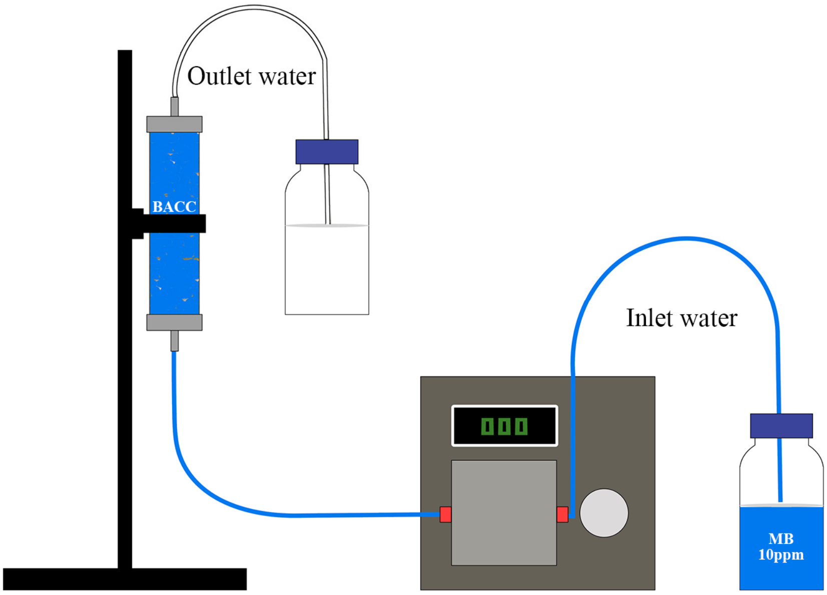

Figure S1. Fixed bed device (MB: 10 mg/L, flow rate 3 mL/min, filter column (10 mL) filled with 0.1 mL BACC and 9.9 mL quartz fine sand, outlet water sample collection time interval of 3 minutes).

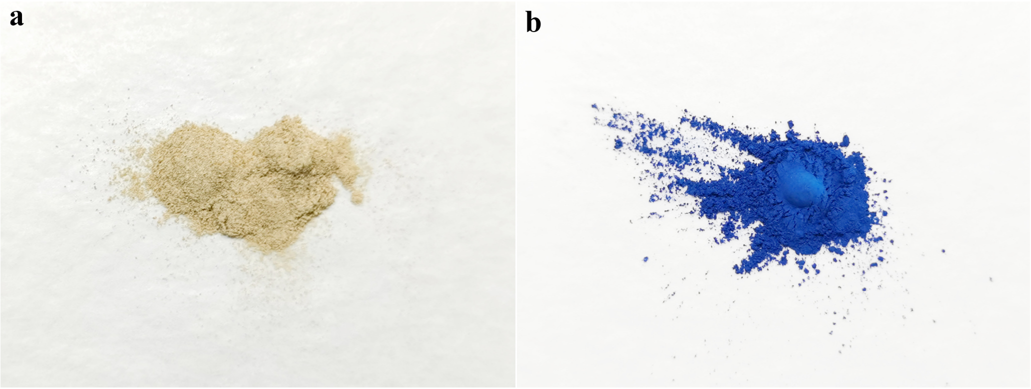

Figure S2. Before- and after-MB adsorption color changes of the BACC sample.

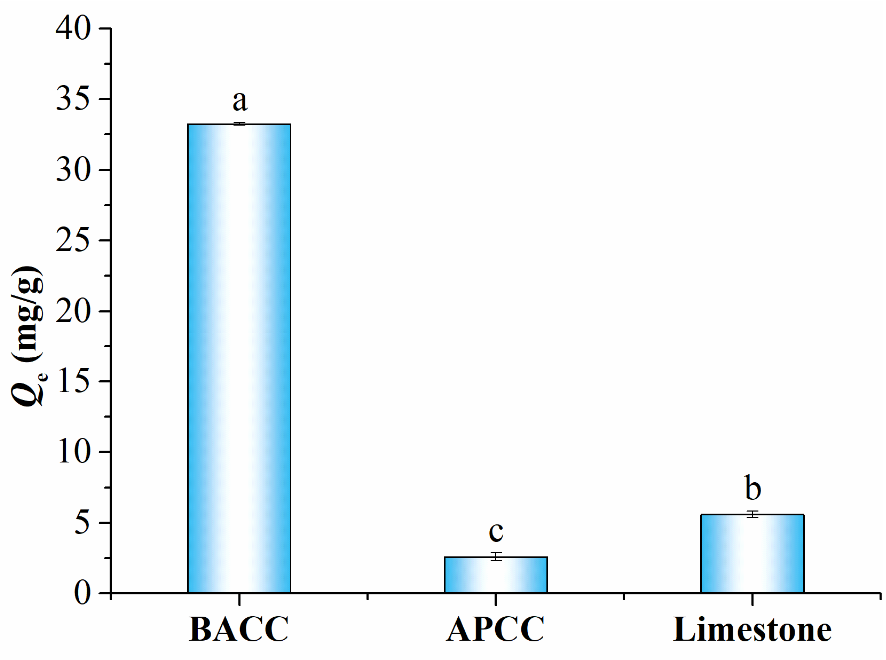

Figure S3. The MB adsorption capacity of BACC, chemically synthesized calcium carbonate (Analytical pure calcium carbonate, APCC), and limestone from the environment. (Bars with different letters indicate statistically significant differences, one-way ANOVA, Duncan's multiple range test, $p < 0.01$). Data represent the mean ± standard deviation (s.d.) from three independent experiments.

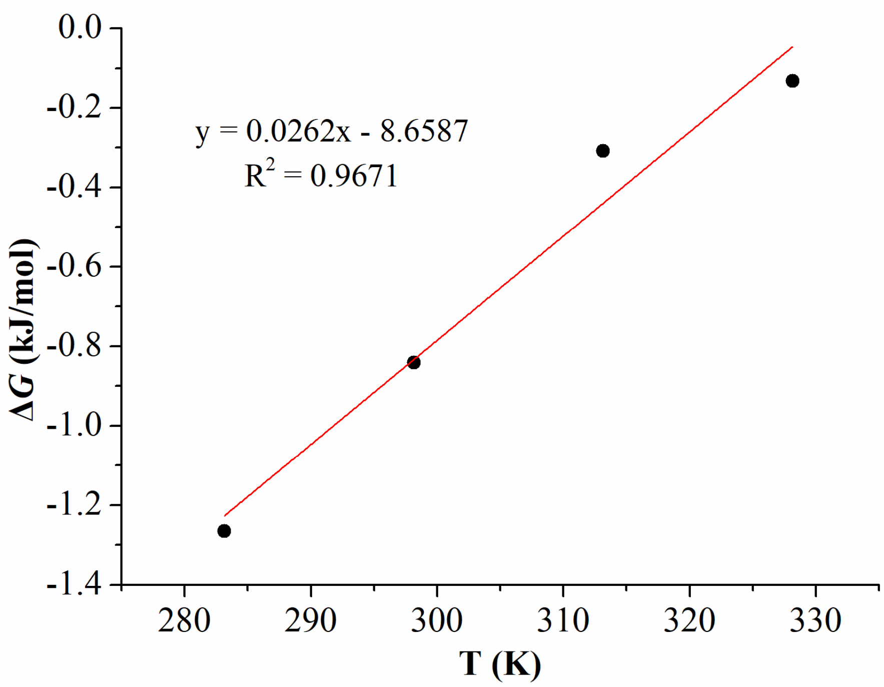

Figure S4. Δ*G* versus temperature for MB adsorption onto BACC.

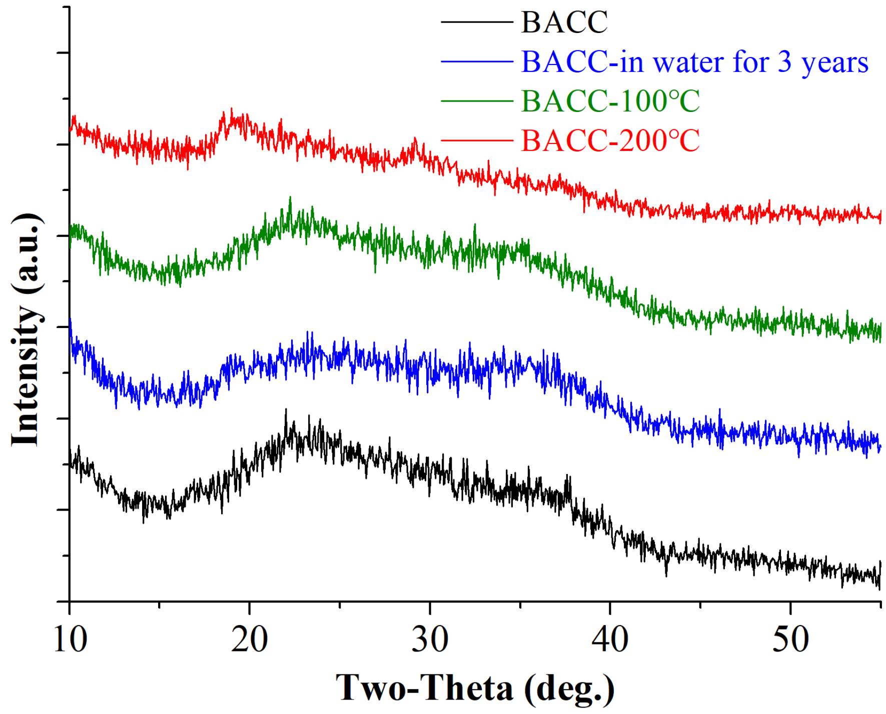

Figure S5. XRD patterns of BACC in water for 3 years and heated at 100 °C, or 200 °C for 2 hours.

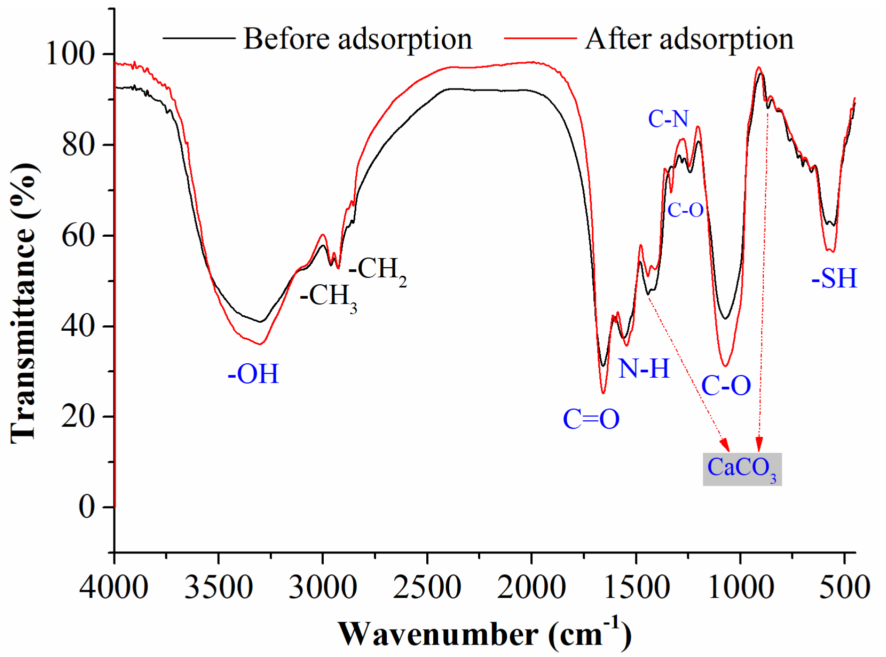

Figure S6. FTIR results of BACC before and after MB adsorption.

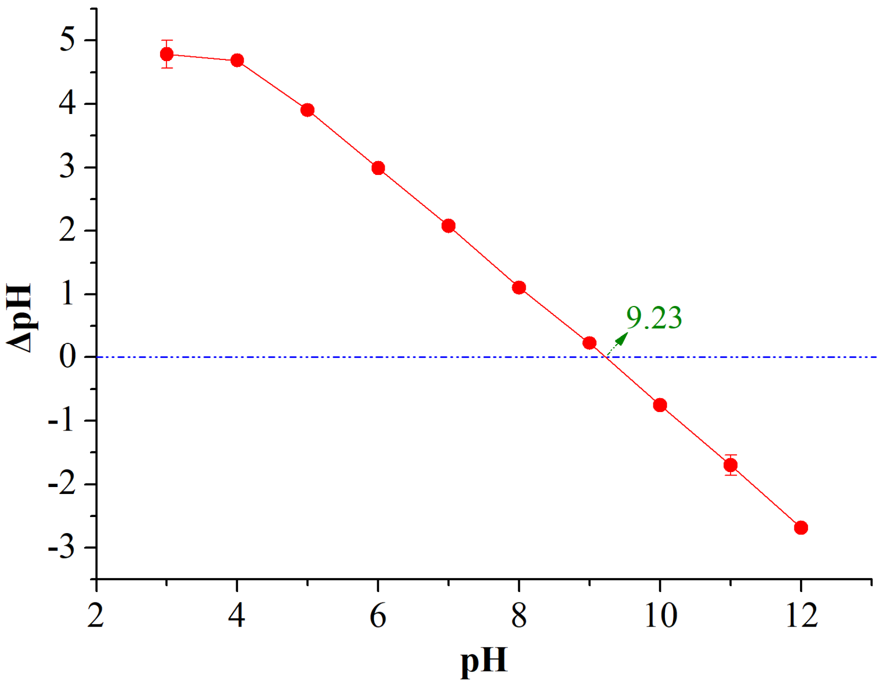

Figure S7. Involvement of electrostatic interaction in the MB adsorption on BACC. ($\Delta pH = pH_e - pH_0$, pH_e is the equilibrium pH value; pH_0 is the initial pH value). Data represent the mean ± standard deviation (s.d.) from three independent experiments.

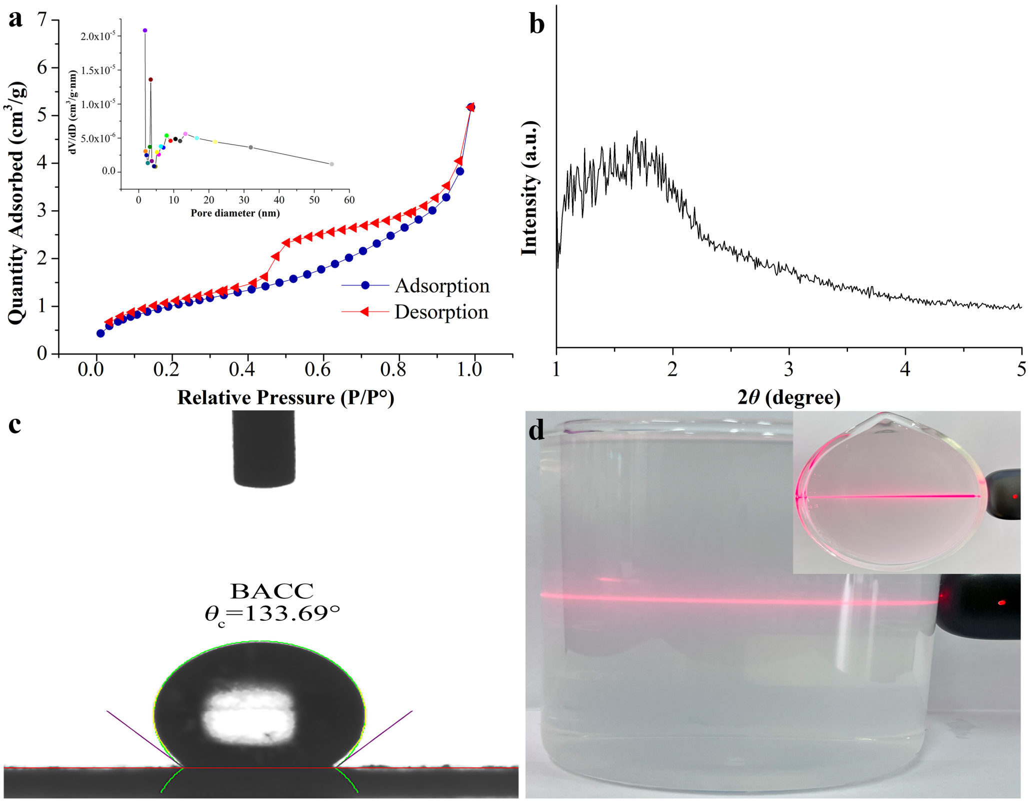

Figure S8. The N_2-sorption isotherms and the pore size distribution (inset) (a), small angle XRD pattern (b), contact angle (c), and Tyndall phenomenon (d) of tested BACC.

Table S1. A comparison of the Q_m values obtained for MB.

Adsorbent	Q_m (mg/g)	Ref.
Kaolin	52.76	[78]
Bentonite	121.51	[79]
Clay	6.33	[80]
Oyster shell	5.51	[81]
Lignin-chitosan extruded pellets	36.25	[82]
Chaff	20.30	[83]
Activated carbon	48.30	[84]
Graphite	41.67	[85]
Platanus orientalis leaf	114.94	[86]
Carbon nanotubes	46.20	[87
Magnetic graphene oxide	232.56	[88]
Peanut hull	108.6	[89]
Potato stem powder	41.6	[90]
C@CS@ MTN	325	[91]
Fe-Mn binary oxide nanoparticles	72.32	[92]
Calcite	118.8	[93]
KOH-activated carbon	546.8	[34]
Graphene oxide/magnesium oxide nanocomposites	833	[35]
BACC	494.86	This work

Table S2. Thermodynamic parameters for MB adsorption on BACC

T (K)	Ln K_c	ΔG (kJ/mol)
283.15	0.5378	-1.2661
298.15	0.3395	-0.8417
313.15	0.1182	-0.3078
328.15	0.0483	-0.1318
ΔH (kJ/mol)	-8.7538	
ΔS (J/mol·K)	-26.5574	

Table S3. Kinetic constants of PFO and PSO models for the MB adsorption on BACC.

Models	Parameters	MB
PFO model	K_1 (min^{-1})	1.6848
	$Q_{e1, cal}$ (mg/g)	63.65
	R^2	0.8796
PSO model	K_2 (g·(mg·min)$^{-1}$)	0.0664
	$Q_{e2, cal}$ (mg/g)	64.07
	R^2	0.9333
Experimental Q_e (mg/g)		65.31

Table S4. The K_2 values comparison of a PSO kinetic model for MB.

Adsorbent	K_2 (g·(mg·min)$^{-1}$)	Ref.
Kaolin	0.6310	[94]
Graphite	0.1798	[85]
Prosopis Juliflora Stem Carbon	0.1297	[95]
Magnetic activated biochar	5.4040	[96]
Defatted Carica papaya seeds	1.3300	[36]
Fe-Mn binary oxide nanoparticles	0.2105	[92]
Potato stem powder	0.7500	[90]
BACC	0.0664	This work

Table S5. Fitting parameters of ID model for adsorption of MB onto BACC.

Experimental	Stage-1			Stage-2			Stage-3		
Q_e(mg/g)	C_1	K_{id1}	R^2	C_2	K_{id2}	R^2	C_3	K_{id3}	R^2
65.31	43.47	8.38	1	61.67	0.16	0.8852	64.05	0.03	0.5467
